# Quality of life in dogs with idiopathic epilepsy and their owners with an emphasis on breed—A pilot study

**DOI:** 10.3389/fvets.2022.1107315

**Published:** 2023-01-11

**Authors:** Maud F. N. Hamers, Marta Plonek, Sofie F. M. Bhatti, Niklas Bergknut, M. Montserrat Diaz Espineira, Koen M. Santifort, Paul J. J. Mandigers

**Affiliations:** ^1^Department of Clinical Sciences, Faculty of Veterinary Medicine, Utrecht University, Utrecht, Netherlands; ^2^Evidensia Referral Hospital, Arnhem, Netherlands; ^3^Small Animal Department, Faculty of Veterinary Medicine, Ghent University, Merelbeke, Belgium; ^4^Evidensia Referral Hospital, Waalwijk, Netherlands

**Keywords:** idiopathic epilepsy, dog, breed, impact, quality of life, owner, treatment

## Abstract

Epilepsy in dogs is a common chronic and serious disorder and may have an impact on the quality of life of the owners as well as the dogs themselves. The aim of this pilot study was to investigate the QoL score of dogs suffering from idiopathic epilepsy and their owners and if possible, investigate whether a breed specific difference exists. Owners, either Dutch or Belgium, were asked to participate in a web based SurveyMonkey questionnaire. A total of 402 questionnaires representing 402 dogs with epilepsy were suitable for further analysis. Of the 402 dogs, 253 were males and 149 were females. Ninety-nine different breeds were represented. Fourteen breeds (177 dogs in total) were used to calculate breed specific scores; Australian Shepherd (*n* = 8), Beagle (*n* = 7), Belgian Tervuren dog (*n* = 9), Belgian Groenendaeler dog (*n* = 8), Border Collie (*n* = 38), Chihuahua (*n* = 9), Dachshund (*n* = 13), Drentsche Patrijshond (a Dutch partridge dog) (*n* = 14), French Bulldog (*n* = 12), Golden Retriever (*n* = 17), Labrador Retriever (*n* = 18), and Rottweiler (*n* = 12). For the Border Collie, there was a statistically significant correlation between “epilepsy related death,” the severity of the seizures (*p* < 0.001) and cluster seizures (*p* < 0.001). The quality of life of the Border Collie was scored lower compared to all other dogs (*p* = 0.02). There were three breeds that had a minimal decrease in the overall quality of life score compared to all other dogs: the Chihuahua (*p* = 0.03), Dachshund (*p* = 0.001), and Golden retriever (*p* = 0.01). The score for “caring for my epileptic dog decreases my own QoL” was high for the Border Collie, Boxer, French Bulldog, and Rottweiler, but was only found to be statistically significantly higher in the Border Collie (*p* = 0.01). Scores for the Golden Retriever (*p* = 0.04) and Labrador (*p* = 0.006) were lower. In conclusion, this study reports breed specific quality of life scores of dogs with epilepsy and their owners, and underlines that breed by itself, is also an important factor when managing epilepsy in dogs.

## Introduction

Epilepsy is the most common chronic canine neurological syndrome and has serious implication on the quality of life (QoL) of both the dogs and owners (1–4). Determining the etiology of canine epilepsy is important and has been the focus of the International Veterinary Epilepsy Taskforce (IVETF), leading to the creation of manuscripts addressing its classification, terminology, and diagnostics (5, 6).

The prevalence of canine epilepsy is estimated to range from 0.6 to 0.75 percent (7, 8), the majority of which is idiopathic, genetic or presumed genetic (9). There is a clear breed predisposition in hunting dogs, shepherds and molosser dogs (9, 10), and several recent studies focussed on the genetics of epilepsy (11–16). But there is also growing interest into the impact of epilepsy and the use of anti-seizure medication (ASM) on owners and their dogs. The goal of ASM is to reduce the number and severity of the seizures. However, the efficacy of various registered ASM's varies and ranges from 70 to 80% with phenobarbital (17, 18), 60% with bromide (17), and just 50% with imepitoin (19–21). Hence, up to 20–30% of all epileptic dogs do not respond favorably to registered ASM's and require multi-drug treatment with unlicensed ASM's such levetiracetam (22, 23), gabapentin (24) or zonisamide (25). These combined therapies introduce considerable side-effects (18, 26).

Hence, several studies have been published investigating the effect of both epilepsy and ASM treatment on the QoL of dogs and their owner (1, 4, 26, 27). Dogs may show behavioral and cognitive changes such as fear/anxiety, aggression, abnormal perception, inattention, and excitability (3, 28, 29). Not only will this have a negative effect on the QoL of the dog but also of the owner (3, 28).

But not all breeds respond equally to ASM. For instance, the outcome in BCs differs clearly from the general population (30). Up to 50% of all young male BCs are euthanized before the age of two as they do not respond to the ASM's (30, 31). Similar observations have been noted for Australian Shepherds (32), Italian Spinone (33), and Rottweiler (34). When there is a breed specific response to ASM's, there is usually also a breed specific QoL score for both dog and owner.

The aim of this pilot study was to investigate the QoL score of dogs suffering from idiopathic epilepsy and their owners and if possible, investigate whether a breed specific difference exists.

## Materials and methods

Owners, either from the Netherlands or Belgium, were asked to participate in a web based SurveyMonkey questionnaire. Owners were approached using social media, direct e-mailing, adverts on canine epilepsy fora and indirectly by spreading the questionnaire with the help of veterinarians in the Netherlands and of Belgium.

The questionnaire consisted of forty questions, based on earlier questionnaires used to evaluate owner perception of dogs with epilepsy in Ohio and Glasgow (27, 35) and the proposed Quality of Life assessment of Wessmann et al. (2). The questions focussed on general aspects of the dog—including breed, age and gender, the number and type of seizures (focal, generalized or other), effect of therapeutic modalities, side effects, the QoL of the dog, and the QoL of the owner. All questions were accompanied with an explanation of the used medical terms, a website page was available with an explanation in Dutch and examples of a tonic-clonic seizure and focal seizures were made available on YouTube. An example of the questionnaire (translated from Dutch into English) can be found in Appendix 1. The questionnaire contained both open and closed questions and some questions allowed multiple answers. The questions addressing the QoL were scored on a scale of 1 to 10. Two groups of questions were formulated. One group addressed the QoL of the dog, the second group the QoL of the owner.

Questions addressing the QoL of the dog:

- Score the severity of the tonic-clonic (large) seizures on the QoL of your dog (1 = not, 10 = severe)
- Score the severity of the focal (small) seizures on the QoL of your dog (1 = not, 10 is severe)
- Score whether the side effects of ASM are acceptable (1 = no side effects, 10 = severe side-effects)
- Score the decrease in QoL now compared to the moment the dog was still free of seizures (0 = 100% decrease, 100 = no decrease)
- Score the quality of life of your dog (1 = zero QoL, 10 = optimal QoL)

Questions addressing the QoL of the owner:

- The severity of seizures is acceptable to me (1 = not acceptable, 10 = no problem at all)
- I dare to leave my dog alone at home for a while (disagree = 1, strongly agree = 10)
- Caring for a dog with epilepsy is worth the effort (1 = it is not worth the effort, 10 = worth)
- I find it difficult to administer ASM to my dog (1 = no, 10 = yes, very much)
- The costs of the treatment are acceptable (1 = no, 10 = yes, very much)
- Regular visits to the consulting vet/specialist make life difficult (1 = not at all, 10 = yes, extremely)
- In the past 3 months I worried about the frequency of seizures in my dog (1 = not at all, 10 = yes, extremely
- Caring for a dog with epilepsy limits my daily activities and causes a decrease of my own quality of life (1 = no not at all, 10 = yes, extremely)

All completed questionnaires were collected with the online survey development software SurveyMonkey within a timeframe of 2 months. Questionnaires were only analyzed if the dog had at least more than two, idiopathic epileptic seizures as described earlier (5) and the questionnaire had been completed. Owners were advised to contact us if there were questions and, on a voluntary base, could sent us the medical record of their dog. Results were analyzed with version 28 of the IBM SPSS Software and included descriptive statistics, chi-squared tests, *t*-tests and Pearson's correlation tests. Means ± standard deviations were calculated for all dogs and compared with the results of specific breeds. Each breed was compared with the entire group of dogs minus the breed to be tested. Breed scores were only calculated if at least six dogs were of the same breed. Results were considered statistically significant if the *p*-value was < 0.05.

## Results

A total of 622 owners (partly or completely) filled in the questionnaire. Based on the selection criteria, 402 questionnaires were suitable for further analysis.

### Signalment

Of the 402 dogs, 253 were males and 149 were females. Males were overrepresented with statistical significance (*p* = 0.001). Owners of 99 different breeds participated. Fourteen breeds (177 dogs in total) could be used to calculate breed specific scores; Australian Shepherd (*n* = 8), Beagle (*n* = 7), Belgian Tervuren (*n* = 9), Belgian Groenendaeler dog (*n* = 8), Border Collie (*n* = 38), Chihuahua (*n* = 9), Dachshund (*n* = 13), Drentsche Patrijshond (a Dutch partridge dog) (*n* = 14), French Bulldog (*n* = 12), Golden Retriever (*n* = 17), Labrador Retriever (*n* = 18), and Rottweiler (*n* = 12). The remaining 225 dogs were either listed as mixed breeds (*n* = 42) or belonged to various other breeds (183 dogs). A complete list of breeds is available in Appendix 2.

### Type and number of seizures

**Figure 1:**
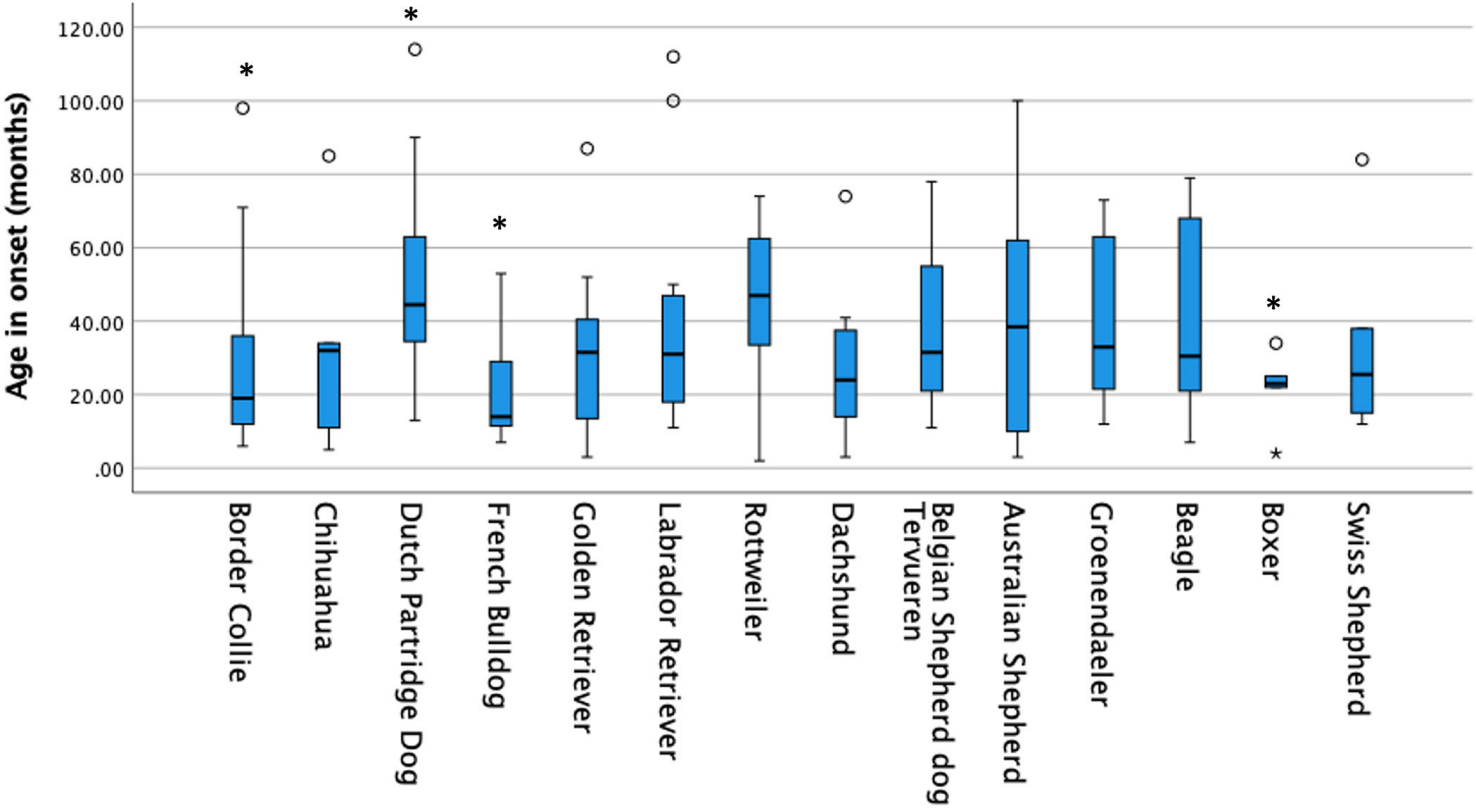
Age in onset for all breeds. The age of onset was for the Border collie, French Bulldog and Boxer significantly lower compared to all other breeds and for the Dutch Partridge Dog higher.

Based on the owners' submissions, 305 of 402 dogs showed mainly generalized tonic-clonic seizures and 97 showed only focal seizures. No association was found between seizure type and breed (*p* = 0.867). On average, for the complete group of 402 dogs, the age of onset was 38 ± 30 months. Three breeds, the Border collie (*p* = 0.003), French Bulldog (*p* = 0.018), and Boxer (*p* = 0.04), showed a statistically significant lower age of seizure onset compared to all other dogs (Table 1 and Figure 1). Compared to all other dogs, the Drentsche Patrijshond showed a statistically significantly higher age of seizure onset (*p* = 0.04). The number of seizures for all dogs was 24 ± 163 over a period of 12 months. Only the Drentsche Patrijshond showed a statistically significant higher number of clusters during a period of 12 months compared to all other dogs (*p* = 0.002) (Table 1). There was no statistically significant difference for the number of status epilepticus events, visits made to the veterinarian or specialist, or number of needed nightly visits, except for the Labrador retriever and Golden retriever. Owners of these two breeds reported statistically significant fewer nightly visits compared to all other owners (*p* < 0.001) (Table 1).

**Table 1:** Descriptive statistics (number, mean ± standard deviation) for all dogs and the 14 selected breeds.

	***n***	**Age of onset in months**	**Number of fits last 12 months**	**Number of clusters last 12 months**	**Number of status last 12 months**	**Number of vet. visits last 12 months**	**Number of evening/night visits last 12 months**
All dogs	402	38 ± 30	24 ± 163	7 ± 29	1.5 ± 6.4	9 ± 6.4	0.7 ± 1.7
Mixed breeds	226	41 ± 33	31 ± 216	7 ± 29	1.2 ± 7.4	9 ± 2.9	0.8 ± 1.8
Australian shepherd	8	40 ± 33	6 ± 7	4 ± 6	0 ± 0	8 ± 2.6	1 ± 1.5
Beagle	7	51 ± 40	21 ± 30	3 ± 4	1 ± 2.9	9 ± 3	0.1 ± 0.4
Border Collie	38	28 ± 21	23 ± 74	8 ± 14	1.2 ± 3.6	9 ± 3	1.2 ± 2.5
Boxer	6	20 ± 11	6 ± 1.6	5 ± 8	2.6 ± 3.2	8 ± 4	0.2 ± 0.4
Chihuahua	9	26 ± 29	13 ± 18	2 ± 3.6	0.3 ± 0.8	10 ± 3	0.1 ± 0.4
Dachshund	13	16 ± 19	16 ± 25	4 ± 9	0 ± 0	10 ± 2	0.6 ± 1
French bulldog	12	20 ± 14	7 ± 7	4 ± 6	0.6 ± 0.9	8 ± 3.6	1.3 ± 1.7
Golden retriever	17	35 ± 28	8 ± 9	2 ± 3.8	0.7 ± 2.4	10 ± 2.2	0.1 ± 0.3
Groenendaeler	8	38 ± 24	7 ± 5	2 ± 4.4	0.4 ± 0.8	9 ± 4	0.9 ± 2.2
Labrador retriever	18	37 ± 28	7 ± 13	3 ± 12	3 ± 12	10 ± 2.7	0.1 ± 0.5
Drentsche Patrijs	14	51 ± 29	9 ± 9	29 ± 99	2 ± 6.6	8.6 ± 2.8	0.8 ± 1.6
Rottweiler	12	45 ± 21	13 ± 9	4 ± 6	0.4 ± 0.8	8 ± 3	0.7 ± 1.3
Swiss shepherd	6	33 ± 27	13 ± 21	9 ± 20	0.2 ± 0.4	7.7 ± 4	0.2 ± 0.4
Tervuren shepherd	9	32 ± 30	55 ± 135	1 ± 2	0.3 ± 0.8	10 ± 2.5	0.2 ± 0.4

The boxes colored lightly gray contain observations that were statistically significant compared to all other dogs.

### Survival time

**Figure 2:**
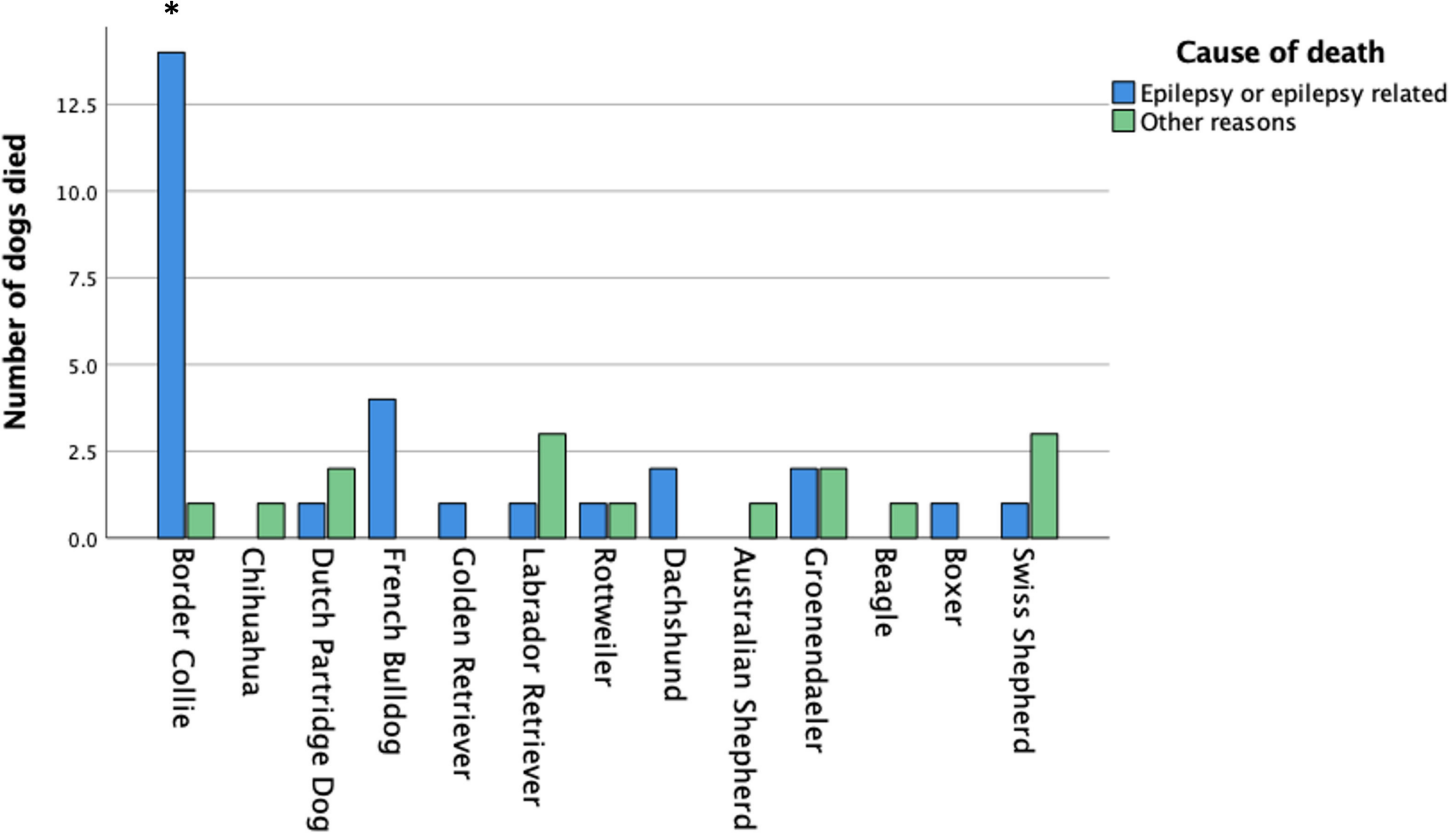
Cause of death for the dogs that had died. Only for the Border collie the ‘epilepsy related death' was statistically significant higher compared to all other dogs.

At the time of the survey, 308 dogs were alive, and 94 dogs were dead. The mean age ± SD of the dogs that were alive (308 dogs) was 6.6 ± 3.2 years. The mean age ± SD at death (94 dogs) was 5.6 ± 4.0 years. Epilepsy-related issues were the cause of death in 71% of the dogs, hence in 29% of the dogs it was not. In the group of Border collies, there was a clear statistically significant association of seizures and death compared to the other breeds (*p* = 0.047; Figure 2). Only in the Border Collie was there a clear, statistically significant correlation of “death,” the severity of the seizures (*p* < 0.001) and clustering (*p* < 0.001). For all other breeds, no statistically significant differences were found.

### Medication

Several ASM strategies were used, either as monotherapy or combination-therapy. Two hundred and eighteen of the 402 dogs were treated with phenobarbital: either in monotherapy (*n* = 92) or in combination with other ASM (*n* = 126). Imepitoin was administered to 85 of the 402 dogs: either in monotherapy (*n* = 39) or in combination with other ASM (*n* = 46). Potassium bromide was used in 135 of 402 dogs: either in monotherapy (*n* = 22) or in combination with other ASM (*n* = 113). Levetiracetam (*n* = 22), gabapentin (*n* = 5) and slow-release phenytoin (*n* = 5) were used less frequently and only in a combination-therapy. Diazepam applied rectally during a seizure event, was used in 78 of 402 dogs. Some owners only used diazepam to stop the seizure and did not give other ASM (*n* = 12). ASMs were not used at all in 60 dogs.

The most used combination-therapies were phenobarbital and potassium bromide (*n* = 77), phenobarbital and imepitoin (*n* = 22), imepitoin and bromide (*n* = 9) and phenobarbital, imepitoin and bromide (*n* = 11). Alternative treatment options were employed in 64 out of 402 cases; 12 dogs were fed special diets; 22 dogs were administered cannabidiol (CBD) oil and 8 CBD/ tetrahydrocannabinol (THC) oil. Other alternative treatments were phytotherapy (*n* = 3) and music therapy (*n* = 1).

Up to 90% of all Border collies (BC) received at least one ASM and up to 47% received at least two different ASM's. Forty-five percent of all Labrador Retrievers and 50% of all Belgian Groenendaeler dogs did not use any ASM. The majority of the Drentsche Patrijshond, the Dachshund and Belgian Tervuren dogs used only one ASM. Bromide was most frequently used in the BC and Australian Shepherd. Although minimal, this difference was not statistically significant (*p* = 0.054).

### Side effects

The most reported side effects were lethargy (*n* = 123), sleepiness (*n* = 117), an increased appetite (*n* = 140) and polydipsia (*n* = 125). The use of monotherapy potassium bromide in dogs had the most side effects. Breed specific scores are discussed in the Questions addressing the QoL of the dog.

### Questions addressing the QoL of the dog

Five questions addressed the QoL of the dog. Interestingly, there was no statistically significant difference in the severity of the TC seizures for any of the 14 breeds compared to all other dogs minus the tested breed (Table 2). There was a statistically significant lower focal seizure severity in the Belgian Groenendaeler dog compared to the other breeds (*p* = 0.04). The owners of the Dachshund (*p* = 0.0003) and Golden retriever (*p* = 0.001) assessed the ASM side effects as very mild compared to all other dogs. ASM side effects were considered more severe compared to all other dogs in the Australian shepherd (*p* = 0.01) and Rottweiler (*p* = 0.007).

**Table 2:** Scores addressing the QoL of the dog for all dogs and the 14 breeds.

	***n***	**Severity TC seizures**	***p*-Value**	**Severity focal seizures**	***p*-Value**	**Side effects acceptable**	***p*-Value**	**Decrease in score**	***p*-Value**	**QoL dog**	***p*-Value**
All dogs	402	6.9 ± 2.7	–	4.7 ± 2.5	–	6.6 ± 3.2	–	66 ± 31	–	7.2 ± 2.7	–
Australian shepherd	8	6.5 ± 3.6	0.32	5.6 ± 2.7	0.43	4.0 ± 2.1	0.01	64 ± 33	0.42	6.0 ± 2.7	0.10
Beagle	7	6.3 ± 2.7	0.26	5.6 ± 2.8	0.43	8.0 ± 2.2	0.12	63 ± 30	0.39	7.3 ± 2.5	0.48
Border Collie	38	7.2 ± 2.8	0.29	4.5 ± 2.4	0.78	6.0 ± 3.3	0.11	60 ± 36	0.12	6.4 ± 2.9	0.02
Boxer	6	8.5 ± 0.8	0.002	5.2 ± 2.0	0.56	6.2 ± 2.8	0.37	62 ± 34	0.36	6.8 ± 2.9	0.36
Chihuahua	9	6.6 ± 3.4	0.33	5.4 ± 2.2	0.43	7.1 ± 2.9	0.32	69 ± 27	0.40	8.9 ± 1.4	0.03
Dachshund	13	5.9 ± 3.1	0.08	4.0 ± 2.7	0.37	8.8 ± 2.4	0.003	82 ± 16	0.001	8.6 ± 1.3	0.001
French bulldog	12	7.7 ± 2.4	0.17	5.7 ± 2.4	0.44	5.8 ± 3.3	0.17	66 ± 37	0.48	6.7 ± 2.8	0.23
Golden retriever	17	5.6 ± 3.0	0.02	3.1 ± 2.2	0.29	8.7 ± 2.0	0.001	78 ± 28	0.06	8.8 ± 1.1	0.01
Groenendaeler	8	7.3 ± 2.6	0.37	2.5 ± 1.7	0.04	7.5 ± 2.5	0.25	63 ± 38	0.39	6.3 ± 3.7	0.17
Labrador retriever	18	6.1 ± 2.6	0.09	3.6 ± 2.2	0.28	6.8 ± 3.7	0.42	78 ± 17	0.006	7.7 ± 2.3	0.23
Drentsche Patrijs	14	7.2 ± 2.6	0.36	4.6 ± 2.1	0.48	6.8 ± 3.1	0.43	59 ± 34	0.17	7.1 ± 2.7	0.45
Rottweiler	12	7.1 ± 2.7	0.43	4.1 ± 2.5	0.38	4.8 ± 2.2	0.007	68 ± 24	0.43	7.5 ± 1.2	0.24
Swiss shepherd	6	7.0 ± 2.7	0.12	4.7 ± 2.5	0.99	5.5 ± 3.1	0.20	60 ± 40	0.31	7.7 ± 2.4	0.34
Tervuren shepherd	9	6.9 ± 1.9	0.47	3.9 ± 3.0	0.31	6.6 ± 3.2	0.32	76 ± 17	0.18	8.3 ± 1.6	0.10

The severity of the tonic-clonic (TC) seizures and focal seizures were measured from 1 (not severe) to 10 (severe). Side effects were scored from 1 (no side effects) to 10 (severe side-effects). The column “decrease in score” showed the decrease in QoL compared to the moment the dog was still free of seizures (0 = 100% decrease, 100 = no decrease). The last column is the final QoL score of the dog (1 = zero QoL, 10 = optimal QoL). Boxes lightly gray contain scores found to be statistically significant.

Owners were also asked to score the decrease in QoL compared to the moment the dog was still seizure free and to score the overall QoL. All dogs had a decrease in QoL after seizure onset, but the Dachshund (*p* = 0.001), Golden retriever (0.06) and Labrador retriever (*p* = 0.006), had the lowest decrease in QoL (Table 2). The Border Collie and Swiss Shepherd had the highest decrease in QoL (average of 40%) compared to all other dogs (average of 34%) but this was not found to be statistically significant. The overall QoL score for all dogs was 7.2 ±2.7. Although the Border Collie, Australian Shepherd and Belgian Groenendaeler dog had an average QoL score of < 6.5, only in the Border Collie was it statistically significantly lower compared to all other dogs (*p* = 0.02). There were three breeds that had a minimal decrease in the overall QoL score compared to all other dogs: the Chihuahua (*p* = 0.03), Dachshund (*p* = 0.001) and Golden retriever (*p* = 0.01) (Table 2).

### Questions addressing the impact of the dog's epilepsy on the owner

Eight questions addressed the impact of the dog's epilepsy on the owner. Overall scores were calculated for all dogs (Table 3). Up to half of all BC owners, compared to all other owners, found it difficult to leave their dog alone at home for a brief period (*p* = 0.028), while Dachshund owners were very confident to leave their dog at home (*p* = 0.005, Table 3). Australian Shepherd owners scored lower compared to other owners on the question asking whether taking care of their dog was worth the effort (*p* = 0.009). Interestingly, Dachshund (*p* < 0.001), Chihuahua (*p* < 0.001), and Rottweiler owners (*p* = 0.007) scored very high: they found that taking care of their dog was very much worth the effort. The overall score for that question, for all owners, was 8.9 ± 2.2 (Table 3). The owners of Dachshunds found the costs of treatment significantly less burdening compared to all other owners (*p* = 0.049). The overall score was 6.4 ± 3. There was no breed specific statistically significant difference concerning the question addressing whether visits to the veterinarian/specialist or ASM administration were troublesome. Veterinary visits were less of an issue for Belgian Groenendaeler dog owners, compared with the other owners (*p* = 0.004). The owners of the Dachshund (*p* = 0.013) and Golden Retriever (*p* = 0.03) reported a lower severity of the seizures compared to all other owners. There was no breed specific difference for “worried during the last 3 months.”

**Table 3:** Scores addressing the impact of the dog's epilepsy on the owner.

	***n***	**Leave the dog at home**	***p*-Value**	**Caring worth the effort**	***p*-Value**	**Costs acceptable**	***p*-** **Value**	**Visits vet/spec**	***p*-** **Value**	**Admin. medication**	***p*-** **Value**	**Severity of seizures**	***p*-** **Value**	**Worried last 3 months**	***p*-** **Value**	**Caring decreases my QoL**	***p*-Value**
All dogs	402	6.3 ± 3.5		8.9 ± 2.2		6.4 ± 3		3 ± 2.9		1.9 ± 1.9		5.2 ± 3.4		5.5 ± 3.7		4.4 ± 3.3	
Australian shepherd	8	5.8 ± 4.2	0.3	7.1 ± 3.4	0.009	6 ± 3.3	0.35	3.9 ± 3.4	0.26	3 ± 2.4	0.43	4.8 ± 3.6	0.34	6.6 ± 3.9	0.2	5 ± 3.5	0.3
Beagle	7	7.3 ± 2.4	0.16	9.4 ± 0.54	0.268	5.3 ± 1.9	0.08	3 ± 2.8	0.43	1.3 ± 0.5	0.2	6.4 ± 3.6	0.17	5.4 ± 3.6	0.47	5.0 ± 3.5	0.3
Border Collie	38	5.3 ± 3.6	0.028	8.4 ± 3	0.127	5.7 ± 3	0.065	4 ± 3.1	0.05	1.8 ± 1.6	0.38	4.3 ± 3.3	0.31	6.6 ± 3.6	0.3	5.6 ± 3.5	0.01
Boxer	6	5.0 ± 3.4	0.32	8.5 ± 1.6	0.32	4.8 ± 2.5	0.10	3.3 ± 3.7	0.46	1.3 ± 0.8	0.24	4.3 ± 2.3	0.2	5.8 ± 4.0	0.42	5.8 ± 3.5	0.14
Chihuahua	9	4.8 ± 3.7	0.09	10 ± 0	< 0.001	7.4 ± 2.2	0.15	3.4 ± 3.1	0.4	1.6 ± 1.1	0.36	5.9 ± 3	0.28	4.3 ± 3.6	0.16	3.9 ± 3.4	0.33
Dachshund	13	8.5 ± 2.7	0.005	9.9 ± 0.3	< 0.001	7.8 ± 2.9	0.049	3.3 ± 3.4	0.45	1.8 ± 2.5	0.48	7.0 ± 2.6	0.013	4.2 ± 3.4	0.09	3.3 ± 3.1	0.12
French Bulldog	12	5.5 ± 3.6	0.21	9.2 ± 0.87	0.3	5.5 ± 2.9	0.14	3.9 ± 3.6	0.2	1.5 ± 0.9	0.24	4.6 ± 3.5	0.25	6.9 ± 3.8	0.1	5.3 ± 3.1	0.18
Golden retriever	17	7.4 ± 3.2	0.1	9.1 ± 2.1	0.4	6.2 ± 3.0	0.37	2.7 ± 2.9	0.24	1.7 ± 1.3	0.36	6.8 ± 3.6	0.03	5.4 ± 4.0	0.42	3.2 ± 2.6	0.04
Groenendaeler	8	7.3 ± 4.0	0.22	7.6 ± 2.5	0.05	5.9 ± 4.3	0.31	1.6 ± 1.1	0.004	1.1 ± 0.4	0.15	5 ± 4.1	0.43	6.1 ± 4.1	0.33	4.0 ± 3.1	0.38
Labrador retriever	18	7.2 ± 3.4	0.12	8.6 ± 3.0	0.24	6.4 ± 3.7	0.49	2.6 ± 2.6	0.17	2.2 ± 2.6	0.25	5.4 ± 3.6	0.39	4.3 ± 3.4	0.08	2.8 ± 2.4	0.006
Drentsche Patrijs	14	6.2 ± 3	0.47	9.1 ± 1.5	0.35	7.3 ± 2.6	0.14	4.3 ± 3.3	0.08	1.6 ± 1.2	0.31	4.8 ± 3.2	0.3	4.7 ± 3.7	0.2	3.6 ± 2.9	0.2
Rottweiler	12	5.7 ± 3.5	0.3	9.7 ± 0.9	0.007	6.7 ± 3.1	0.38	2.3 ± 1.8	0.13	1.7 ± 1.8	0.35	4.8 ± 2.7	0.34	5.8 ± 3	0.4	5.3 ± 3.7	0.18
Swiss shepherd	6	6 ± 4	0.4	9.5 ± 1.2	0.26	7.3 ± 3.2	0.23	2.3 ± 2.8	0.23	1.3 ± 0.8	0.24	6.7 ± 3.0	0.15	3.2 ± 3.5	0.08	3.7 ± 2.7	0.27
Tervuren shepherd	9	5.9 ± 4.1	0.37	9.4 ± 0.9	0.23	6.2 ± 3.1	0.42	3.1 ± 3.1	0.46	1.3 ± 1	0.19	6.7 ± 3.6	0.1	6.7 ± 4.1	0.18	5 ± 3.8	0.29

Questions related to whether they felt confident to leave the dog alone for a while (1 = I cannot, 10 = no problem at all); whether the care itself was worth the effort (1 = it is not worth the effort, 10 = worth); whether the administration of the medication to the dog caused problems (1 = no, 10 = yes, very much); acceptability of the treatment costs (1 = no, 10 = yes, very much); whether the visits to the veterinarian/specialist were an issue (1 = not at all, 10 = yes, very much); acceptability of the severity of seizures for the owner (1 = not acceptable, 10 = no problem at all); whether the owner was worried during the past 3 months about the frequency of seizures of the dog (1 = not at all, 10 = yes, very much), and lastly whether the caring for a dog with epilepsy limited the daily activities and caused a decrease of the QoL score of the owner (1 = no not at all, 10 = yes very much).

Boxes lightly gray contain scores found to be statistically significant.

The score for “caring for my epileptic dog decreases my own QoL” was high for the Border Collie, Boxer, French Bulldog and Rottweiler was found to be statistically significantly lower compared to all other breeds only in the Border Collie (*p* = 0.01). A positive difference was noted for the Golden Retriever (*p* = 0.04) and Labrador (*p* = 0.006) (Table 3).

## Discussion

**Figure 3:**
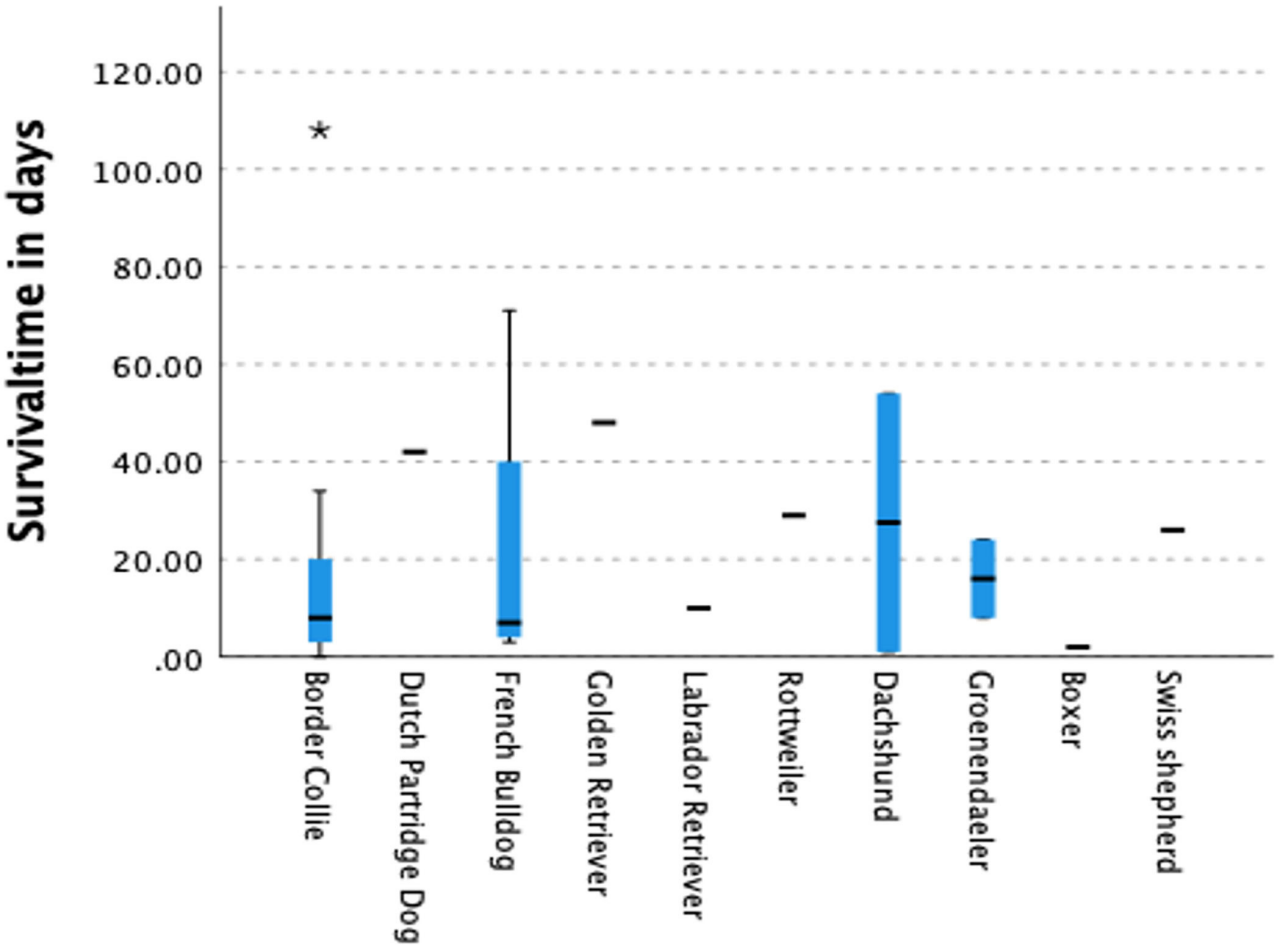
Survival time in days after the onset of the seizures. Only for the Border collie the survival time was statistically significant lower compared to all other dogs. It was also low for the French Bulldog and Boxer although not statistically significant.

The assessment of QoL in epileptic dogs and their owners has been previously studied in various countries (1, 3, 4, 26–28, 36) but limited information is available for breed specific QoL scores. To date, two breed specific studies addressing QoL score have been published. One was performed in 2016 in the Italian Spinone (33) and a second one recently in the Border Collie (30). Furthermore, breed comparisons for QoL of dogs and owners are, to the authors knowledge, limited. The aim of this pilot study was to investigate breed specific QoL scores for dogs suffering from idiopathic epilepsy and their owners. During a period of 2 months, 402 questionnaires were obtained from the owners of several dog breeds. In total, 14 breeds were overrepresented, which enabled a comparison of breed specific QoL scores of both owners and their epileptic dogs. Several interesting breed specific observations were noted. Compared with all breeds/dogs in this pilot study, the breed with the poorest statistically significant outcome appeared to be the Border Collie. This breed suffers not only from early onset seizuring (Figure 1), a low survival rate due to the severity of the seizures and the development of cluster seizures (Figures 2, 3), and up to 90% of all dogs required ASM and no < 47% was on a combination-therapy of ASMs. Sadly enough, this breed also had the lowest, statistically significant, QoL for both dog (Table 2) and owner (Table 3). Similar observations were noted in 2010 by Hulsmeyer et al. and just recently confirmed by our group (31). Wessmann et al. (1) reported that QoL was predominantly influenced by seizure frequency rather than the severity or clustering. Although not statistically significant, this was also the observation in this study. The Belgian Tervuren dog had the highest number of seizures (Table 1) compared to all other breeds/dogs, although this was not coupled with a high owner severity score (Table 3). In this study, the Border collie owners scored the severity of the seizures as high (Table 2).

An analogous poor outcome, like that of the Border Collie, was expected for the Australian Shepherd. An earlier study describing the characteristics of idiopathic epilepsy in this breed (32) was comparable with a similar study in the Border Collie (31). Although the owners of the Australian Shepherds score their dogs' and their own QoL low, it was not statistically significant compared with the other breeds. Surprisingly, the survival outcome in the Australian Shepherd was clearly different to that of the Border Collie (Figure 2). The only clear difference between these two breeds was, in this study, the age of onset which was in the Border collie much lower. But this was in two other breeds also statistically significant: the French Bulldog and the Boxer. The survival time in both breeds was also low, although this difference was not statistically significant. As far as the Border Collie is concerned this observation has just recently been confirmed in another study (30).

The only breed, in this study, with a similar, but not statistically significant, low survival rate was the French Bulldog (Figures 1, 2). Surprisingly, the French Bulldog, Boxer and Border Collie all had a statistically significant early age of seizure onset (Table 1). The number of seizures was low in the French Bulldog and Boxer, but the severity of seizures was scored as high, although this difference was not statistically significant for the French Bulldog. In contrast to the earlier study of Wessmann et al. (1), we found that there is most likely a combination of factors that influence the QoL in these breeds rather than just the number of seizures. Next to the seizure numbers, early age of seizure onset and the severity of the seizures were also predictors of outcome in some breeds. The number of clusters was, except for the Drentsche Patrijshond, apparently not important. The Drentsche Patrijshond, a Dutch hunting dog, had the highest age of onset and the highest number of cluster seizures. However, the owners scored their dog's and their own QoL comparable to that of other dog owners/dogs. Hence, clustering appears to not be a predominant factor for QoL.

The QoL was scored statistically significantly higher for the Chihuahua, Dachshund and Golden retriever than the other breeds. The QoL score of the owners of Golden retrievers and Labrador retrievers was least affected by the epilepsy (Table 3). Owners of the Chihuahua, Dachshund and the Drentsche Patrijshond also reported an overall low impact of their dog's seizures on their QoL. Surprisingly, the number of seizures was high in the Chihuahua and Dachshund compared to the Labrador retriever, Golden retriever and Drentsche Patrijshond (Table 2). However, the number of seizures was not the predominant factor influencing the QoL score.

The effects of ASM side effects on QoL were also assessed. The acceptability of ASM side effects was statistically significantly higher in the Chihuahua and Golden retriever (Table 2). In other breeds, the ASM side effects appear to have no major influence on the QoL score of the dog. The same could be concluded for the number of visits to the consulting veterinarian/specialist (Table 1). There were statistically significantly fewer evening or night emergency visits in the Golden retriever and Labrador retriever than in other breeds, while the most emergency visits were reported in the Border Collie and French Bulldog (Table 2).

In the Border Collie, French Bulldog, and Boxer, costs of care were scored as least acceptable, although this was not statistically significant (Table 3). These costs were not due to the number of needed (evening/nightly) veterinary visits, as these were comparable to all other breeds/dogs (Table 1) but the owners scored them as higher compared to the other breeds/dogs (Table 3).

An important factor that influences the QoL scores of an owner is the ability to lead a normal life. The owners of Border Collies scored being able to leave the dog alone statistically significantly lower compared to other breeds, and this question also scored low in breeds such as the Boxer and French Bulldog. Owners of these breeds scored their own QoL low. This question also scored low for the Chihuahua owners, although this breed had the highest QoL score (Table 2).

This seems to suggest that there is not only a breed specific, but also an owner-specific impact on QoL. Owners choose their dogs for various reasons. A Border Collie owner may plan to use their dog for other tasks than a Chihuahua owner. Expectations, or disappointments, will have a clear influence on the outcome. Owners usually choose their dogs based on breed-specific traits, which may be strongly impacted by epilepsy-related factors. It is interesting to note that owners of some breeds accept disease-related changes more easily than others. This warrants further research.

This study has limitations. A significant limitation of any on-line questionnaire is that the population studied is self-selected. There is a possibility that owners with good QoL and good seizure control are underrepresented in the study. As there is a clear breed difference we expect that this bias is minimal. And although we were able to collect 402 questionnaires, the number of questionnaires obtained were low for five of the 14 breeds. Obtaining more filled-in questionnaires may have allowed a broader breed-specific analysis. However, seven breeds were clearly overrepresented to provide first insights into breed specific QoL scores for both dogs and owners.

## Conclusions

This is the first study reporting breed-specific differences in the perceived QoL of dogs with idiopathic epilepsy and their owners. In this study, Border Collies and their owners had statistically significantly lower QoL scores than other dog breeds. What influences the QoL score differs for each breed, although the age of seizure onset, the number of seizures and their severity have a negative influence on the perceived QoL. Owners of Labrador retrievers and Golden retrievers reported a minimal impact of the seizures on their pet's QoL, with Chihuahua and Dachshund owners also reporting an overall good QoL of their dogs. Costs of medical care, veterinary visits, and the inability to leave the dog alone may be of negative influence in breeds that already have a negative outcome.

## Data availability statement

The raw data supporting the conclusions of this article will be made available by the authors, without undue reservation.

## Ethics statement

Ethical review and approval was not required for the animal study because it is a questionnaire web-based study. Written informed consent was obtained from the owners for the participation of their animals in this study.

## Author contributions

PM was responsible for the study conception and data collection in co-operation with co-authors. Statistical analysis, data analysis, and manuscript writing were performed by MH and PM. PM supervised data analysis and manuscript editing, in co-operation with MP, MD, SB, and KS. All authors contributed to the article and approved the submitted version.
