# Supplementary material for: Quality of life in dogs with idiopathic epilepsy and their owners with an emphasis on breed—A pilot study
Source: Front Vet Sci. 2023 Jan 11;9:1107315. doi: 10.3389/fvets.2022.1107315 (PMC9874297; doi:10.3389/fvets.2022.1107315)
Supplement: Supplementary file 1 [file Data_Sheet_1.pdf]

TRANSLATED SURVEY

Dear sir / Madam,

Thank you for completing this survey. This survey examines the experiences of dog owners whose dog suffers from epilepsy, and the impact of caring for a dog with epilepsy. Hopefully, the answers will help us better support you in treating a dog with epilepsy. This survey will take 15 to 30 minutes to complete.

Thank you again for completing this survey.

Maud Hamers (veterinary medicine student)
Dr. Paul Mandigers (veterinary neurology specialist)

If you have any questions and/or comments, please post them on the last page of this survey or contact us directly using email address. We will contact you.

If you can send us your dog's medical record as well it is much appreciated. upload button

QUESTIONS

1. What is your dog's name?
2. What is the birthday of your dog?
3. What is the gender of your dog? Male/Female
4. What kind of breed / type is your dog?
5. When did you see your dog's first seizure? If you do not remember the exact date, please give an approximate date.
6. Has your dog experienced more than one seizure? Yes/No
7. Do you know what causes your dog's seizures? Only one answer is possible
   - Proven genetically
   - Idiopathic (i.e. we don't know the cause, often presumably genetic)
   - Metabolic / reactive (= the cause is outside the brain, such as a heart, liver or kidney problem)
   - Structural / secondary (= the cause is in the brain, such as trauma, inflammation, tumor)
   - Other (please give further explanation)

(If you are in doubt, contact us first. Additional information can be found on the following website: https://www.veterinair-neuroloog.nl/ziektes/epilepsie)

8. What type of seizures does your dog typically exhibit?
   - Generalized tonic-clonic seizures (major) For an example see this you tube film: https://www.youtube.com/watch?v=6ticKGNgojw
   - Focal or partial seizures (minor) For an example see this you tube film: https://youtu.be/AZhL9-_MvEM
   - Other, namely...
9. Who is the primary caretaker of your dog?
   - (First-line) practitioner
   - Specialist internal medicine
   - Veterinary neurology specialist

10. If you went to a specialist: how did you met the specialist?
   - I arranged this myself
   - I did this on the advice of my vet
   Differently, namely
11. Is your dog still alive? Yes/No
12. If your dog has died: at what age did your dog pass away?
13. What was the cause of death of your dog?
   - The reason for its death was the epilepsy.
   - Differently, namely
14. How many seizures has your dog had in the last few months? Count the clusters* as an attack.
   - For the last 12 months my dog has had _____seizures
   - For the last 6 months my dog has been having _____seizures
   - For the last 3 months my dog has been having _____seizures
   - For the last month my dog has been having ______seizures

* A cluster means: attacks that follow one another (within 24 hours)

15. How many clusters has your dog had in the last few months? A cluster is two or more seizures during a timeframe of 24 hours. If your dog had for instance 3 seizures on a Friday and 4 on a Saturday it counts as two clusters
   - For the last 12 months my dog has had _____clusters
   - For the last 6 months my dog has been having _____clusters
   - For the last 3 months my dog has been having _____clusters
   - For the last month my dog has been having ______clusters
15. How often have you had a status epilepticus* in the last few months?
   - The last 12 months there has been _____ status epileptic
   - The last 6 months there has been _____ status epileptic
   - The last 3 months there has been ______ status epileptic
   - In the last month there has been ______ status epilepticus

* Status epilepticus means that the seizures follow each other within a very short time, with no recovery phase

16. I rate the severity of the tonic-clonic (major) seizures as: (with 1 not serious and 10 very serious)

    Drag the slider to the desired position

17. I rate the severity of the focal (minor) seizures as: (with 1 not severe and 10 very severe)

    Drag the slider to the desired position

18. I can predict from the behavior of the dog when an attack is coming; my dog shows a changed behavior some time before the seizure (with 1 disagree and 10 strongly agree)

    Drag the slider to the desired position

19. How often does your dog go, for a check-up, to your vet or specialist?
   - Once a week
   - Once every 2 weeks
   - Once every 3 weeks
   - Once a month
   - Once every 2 months
   - Once every 3 months
   - Once every 4 months
   - Once every 5 months

- Once every 6 months
- Once a year
Otherwise, namely...

20. In the past few months, how often did you had to go to a vet in the evenings or on weekends because of your dog's epilepsy?
    - Over the last 12 months… times
    - The past 6 months... times
    - The past 3 months ... times
    - In the past month ... times
21. What medication is/was your dog using? (Multiple answers possible)
    - Phenobarbital (Phenoral®)
    - Phenytoin (Epitard®)
    - Imepitoin (Pexion®)
    - Potassium Bromide (Epikal®, Libromide®)
    - Diazepam, Valium, Stesolid®
    - Gabapentin
    - Levetiracitam (Keppra®)
    - No medication

    Otherwise, namely...
22. What kind of side effects of the medication do you see in your dog? (Multiple answers are possible)
    - Lethargy
    - Sleeping more
    - Restlessness
    - Irritable
    - Being unsteady/uncoordinated
    - Increase in appetite
    - Weight gain
    - Vomiting
    - Diarrhea
    - Defecating in the house
    - Drinking more
    - More urinating
    - Coughing
    - Rash

    Otherwise, namely...
23. Is your dog using any alternative treatments? Yes/No
24. What kind of alternative treatments are you using? (Multiple answers possible)
    - phytotherapy
    - CBD oil
    - CBD/THC oil
    - MCT oil
    - Special diet
    - Music therapy

    Otherwise, namely...

25. Looking back to the period before your dog developed epilepsy, if that period was 100% correct. How do you score your dog now?

Drag the slider to the desired position

(Drag to 0 is it declined to the absolute minimum, 100 means no change at all)

26. In the last 3 months I have been concerned about the frequency of my dog's seizures.

Drag the slider to the desired position.

(If you disagree put it on 1 and if you strongly agree on 10)

27. The severity of my dog's seizures is acceptable to me.

Drag the slider to the desired position.

(If you disagree put it on 1 and if you strongly agree on 10)

28. I dare to leave my dog at home alone.

Drag the slider to the desired position.

(If you disagree put it on 1 and if you strongly agree on 10)

29. Taking care of my dog with epilepsy limits my daily activities; it causes a decrease in my own quality of life.

Drag the slider to the desired position.

(If you disagree put it on 1 and if you strongly agree on 10)

30. Taking care of my dog with epilepsy is worth it.

Drag the slider to the desired position.

(If you disagree put it on 1 and if you strongly agree on 10)

31. The administration of the medication to my dog causes problems.

Drag the slider to the desired position.

(If you disagree put it on 1 and if you strongly agree on 10)

32. The side effects of the medication on my dog are acceptable to me.

Drag the slider to the desired position.

(If you disagree put it on 1 and if you strongly agree on 10)

33. The cost of epilepsy treatment is acceptable to me.

Drag the slider to the desired position.

(If you disagree put it on 1 and if you strongly agree on 10)

34. Are regular visits to your vet or specialist for a consultation a problem for you? (for example, for check-ups, research of the blood concentrations of the medication)?

Drag the slider to the desired position.

(If you disagree put it on 1 and if you strongly agree on 10)

35. Score your dog's quality of life.

Drag the slider to the desired position.

(If you score it very low put it on 1 and if you think it is perfect on 10)

36. Which stage of epilepsy do you consider to be the biggest drain on your dog's quality of life?

- starting phase / prodromal phase (This is a phase of abnormal behavior, possibly accompanied by vomiting; it takes a few seconds to days. It is the phase prior to the actual seizure).
- The attack / ictus / seizure (This is a phase of loss of consciousness and cramps; this may be accompanied by barking, drooling, urinating, and defecating; this takes seconds to minutes).
- The recovery phase / post-ictal phase (This is a stage where the dog is sometimes unable to walk, sleepy, restless, and possibly aggressive; it takes seconds to weeks).

- No specific phase

37. Unfortunately, it is very difficult to obtain complete freedom of attack. But what do you consider reasonably acceptable?
    - One attack per week
    - One attack per 2 weeks
    - One attack per 3 weeks
    - One attack per month
    - One attack every 2 months
    - One attack every 3 months
    - One attack every 4 months
    - One attack every 5 months
    - One attack every 6 months
    - One attack per year
    - Free from attacks

38. Thank you very much for your participation in this research. If you are interested in the results of the survey, please enter your email address below and you will receive the final survey results.

If you have any (additional) questions and/or comments, please post them here.
