# Supplementary material for: Quality of life in dogs with idiopathic epilepsy and their owners with an emphasis on breed—A pilot study
Source: Front Vet Sci. 2023 Jan 11;9:1107315. doi: 10.3389/fvets.2022.1107315 (PMC9874297; doi:10.3389/fvets.2022.1107315)
Supplement: Supplementary file 2 [file Data_Sheet_2.pdf]

Addendum 2: Included breeds

Breed	Frequency	Percentage
American bulldog	1	0.25
American Staffordshire terrier	6	1.49
Australian Shepherd	8	1.99
Basset Fauve de Bretagne	2	0.50
Bavarian Mountain Hound	2	0.50
Beagle	7	1.75
Beauceron	1	0.25
Belgian Shepherd Dog Tervueren	9	2.24
Belgian Shepherd Dog Tervueren x	1	0.25
Berger Blanc Suisse	6	1.49
Bernese Mountain Dog	2	0.50
Biewer Yorkshire Terrier	1	0.25
Boerboel	1	0.25
Bohemian Shepherd	1	0.25
Border Collie	38	9.45
Border Collie x Beagle	1	0.25
Border Collie x Shepherd dog	1	0.25
Border Collie x Shetland Sheepdog	1	0.25
Bouvier	3	0.75
Boxer	6	1.49
Briard	1	0.25
Cane corso	3	0.75
Cardigan Welsh Corgi	2	0.50
Cavalier King Charles Spaniel	1	0.25
Chihuahua	9	2.24
Chihuahua crossbreed	1	0.25
Crossbreed	42	10.45
Crossbreed boomer	8	1.99
Czechoslovakian Wolfdog	1	0.25
Dachshund	13	3.23
Dobermann	3	0.75
Dogue de Bordeaux	1	0.25
Dutch Partridge Dog	14	3.48
Dutch Sheepdog	1	0.25
Dutch Shepherd	4	1.00
Dutch Smoushond	1	0.25
English Bull Terrier	1	0.25
English Bulldog	1	0.25
English Cocker Spaniel	1	0.25
English Pointer	1	0.25
English Springer Spaniel	1	0.25
Flat-Coated Retriever	1	0.25

French Bulldog	12	2.99
German pointer	3	0.75
German Shepherd	2	0.50
Giant Schnauzer	1	0.25
Golden Retriever	17	4.23
Gordon Setter	1	0.25
Grand Basset Griffon Vendéen	2	0.50
Great Dane	1	0.25
Greater Swiss Mountain Dog	2	0.50
Groenendael dog	8	1.99
Havanese	2	0.50
Husky	3	0.75
Irish Setter	5	1.24
Irish Wolfhound	1	0.25
Jack Russell terrier	5	1.24
Keeshond	2	0.50
Keeshond – large	1	0.25
King Charles spaniel	3	0.75
Kooikerhondje	2	0.50
Labradoodle	3	0.75
Labradoodle - Australian	1	0.25
Labrador Retriever	18	4.48
Labrador Retriever x	2	0.50
Lagotto Romagnolo	1	0.25
Lhasa Apso	2	0.50
Malinois dog	1	0.25
Maltese	4	1.00
Miniature Pinscher	1	0.25
Nova Scotia Duck Tolling Retriever	2	0.50
Old English Bulldog	4	1.00
Old German Shepherd Dog	1	0.25
Papillon	1	0.25
Peruvian Hairless Dog	1	0.25
Pit Bull	1	0.25
Podenco	3	0.75
Polish Tatra Sheepdog	1	0.25
Pomeranian	3	0.75
Poodle	4	1.00
Pug	1	0.25
Pyrenean Shepherd x	1	0.25
Rat Terrier	3	0.75
Rhodesian Ridgeback	7	1.74
Rhodesian Ridgeback x	1	0.25
Rottweiler	12	2.99
Saarloos wolfhound	3	0.75
Schipperke	1	0.25
Scotch Collie – Short Haired	2	0.50

Shar Pei	2	0.50
Shepherd dog x	4	1.00
Shetland Sheepdog	1	0.25
Shiba inu	1	0.25
Shih Tzu	1	0.25
Small Munsterlander	1	0.25
Soft Coated Wheaten Terrier	1	0.25
South Russian Ovtcharka	1	0.25
Spanish Greyhound	1	0.25
St. Bernard	2	0.50
Stabyhoun	4	1.00
Stabyhoun x	2	0.50
Stabyhoun x Border Collie	2	0.50
Stabyhoun x Small Munsterlander	1	0.25
Stabyhoun x Wetterhoun	1	0.25
Staffordshire Bull Terrier	2	0.50
Tibetan Terrier	3	0.75
Vizsla	1	0.25
Weimaraner	1	0.25
Welsh Springer Spaniel	2	0.50
Wirehaired Vizsla	1	0.25
Total	402	100.0
